# Needs for Increased Awareness of Gastrointestinal Manifestations in Patients With Human Inborn Errors of Immunity

**DOI:** 10.3389/fimmu.2021.698721

**Published:** 2021-08-12

**Authors:** Eun Sil Kim, Dongsub Kim, Yoonsun Yoon, Yiyoung Kwon, Sangwoo Park, Jihyun Kim, Kang Mo Ahn, Soomin Ahn, Yon Ho Choe, Yae-Jean Kim, Mi Jin Kim

**Affiliations:** ^1^Department of Pediatrics, Samsung Medical Center, Sungkyunkwan University School of Medicine, Seoul, South Korea; ^2^Department of Pediatrics, Kyungpook National University Hospital, School of Medicine, Kyungpook National University School of Medicine, Daegu, South Korea; ^3^Department of Pediatrics, Korea University Guro Hospital, College of Medicine, Korea University, Seoul, South Korea; ^4^Department of Pathology, Samsung Medical Center, Sungkyunkwan University School of Medicine, Seoul, South Korea

**Keywords:** inborn errors of immunity, primary immunodeficiencies, gastrointestinal, endoscopy, malignancy, inflammatory bowel disease

## Abstract

The gastrointestinal (GI) tract is frequently affected by inborn errors of immunity (IEI), and GI manifestations can be present in IEI patients before a diagnosis is confirmed. We aimed to investigate clinical features, endoscopic and histopathologic findings in IEI patients. This was a retrospective cohort study conducted from 1995 to 2020. Eligible patients were diagnosed with IEI and had GI manifestations that were enough to require endoscopies. IEI was classified according to the International Union of Immunological Societies classification. Of 165 patients with IEI, 55 (33.3%) had GI manifestations, and 19 (11.5%) underwent endoscopy. Among those 19 patients, nine (47.4%) initially presented with GI manifestations. Thirteen patients (68.4%) were male, and the mean age of patients 11.5 ± 7.9 years (range, 0.6 – 26.6) when they were consulted and evaluated with endoscopy. The most common type of IEI with severe GI symptoms was “Disease of immune dysregulation” (31.6%) followed by “Phagocyte defects” (26.3%), according to the International Union of Immunological Societies classification criteria. Patients had variable GI symptoms such as chronic diarrhea (68.4%), hematochezia (36.8%), abdominal pain (31.6%), perianal disease (10.5%), and recurrent oral ulcers (10.5%). During the follow-up period, three patients developed GI tract neoplasms (early gastric carcinoma, mucosa associated lymphoid tissue lymphoma of colon, and colonic tubular adenoma, 15.8%), and 12 patients (63.2%) were diagnosed with inflammatory bowel disease (IBD)-like colitis. Investigating immunodeficiency in patients with atypical GI symptoms can provide an opportunity for correct diagnosis and appropriate disease-specific therapy. Gastroenterologists and immunologists should consider endoscopy when atypical GI manifestations appear in IEI patients to determine if IBD-like colitis or neoplasms including premalignant and malignant lesions have developed. Also, if physicians in various fields are better educated about IEI-specific complications, early diagnosis and disease-specific treatment for IEI will be made possible.

## Introduction

Human inborn errors of immunity (IEI) are a heterogeneous and increasing set of more than 350 genetic disorders affecting the development and/or the function of various components of the innate and adaptive immune system (1). Although respiratory symptoms seem to be the most common manifestation of IEI, the gastrointestinal (GI) system is the second most common site of complications.

Recent studies revealed that the prevalence of GI manifestations in patients with IEI varies from 5% to 50% and depends on the specific immunodeficiencies in each patient (2). This is partially because the gut-associated lymphoid tissue is the largest lymphoid organ in the body and is thus frequently affected by states of immune deficiency (2, 3). In addition, the mucosal immune system of the GI tract is carefully regulated to maintain homeostasis in the face of exposure to bacterial antigens, viruses, fungi, and dietary antigens, all of which exist in close proximity to a large reservoir of immune cells, such as lymphocytes, macrophages, and dendritic cells. Impairment of the regulatory mechanisms that maintain homeostasis between active immunity and tolerance in the GI tract may cause mucosal inflammation and damage (4). Therefore, the GI tract, which contains an abundance of lymphoid tissue, is frequently affected in IEI, and GI manifestations can be present in patients with IEI before diagnosis.

The GI manifestations of IEI are divided into 4 groups: infection, malignancy, inflammation, and autoimmunity (5–7). The GI symptoms in patients with IEI may mimic those of other GI diseases, such as inflammatory bowel disease (IBD), although the pathophysiology, treatment strategy and response to conventional treatment are distinct. Thus, patients presenting with atypical GI disease and/or failure to respond to conventional therapy should be evaluated for the possibility of underlying IEI and appropriate treatment should be initiated.

The GI manifestations of IEI have not received much attention from physicians in various fields. We aimed to investigate the characteristics of GI symptoms and the endoscopic and histopathologic findings in IEI patients who had been evaluated with endoscopies.

## Materials and Methods

**Figure 1:**
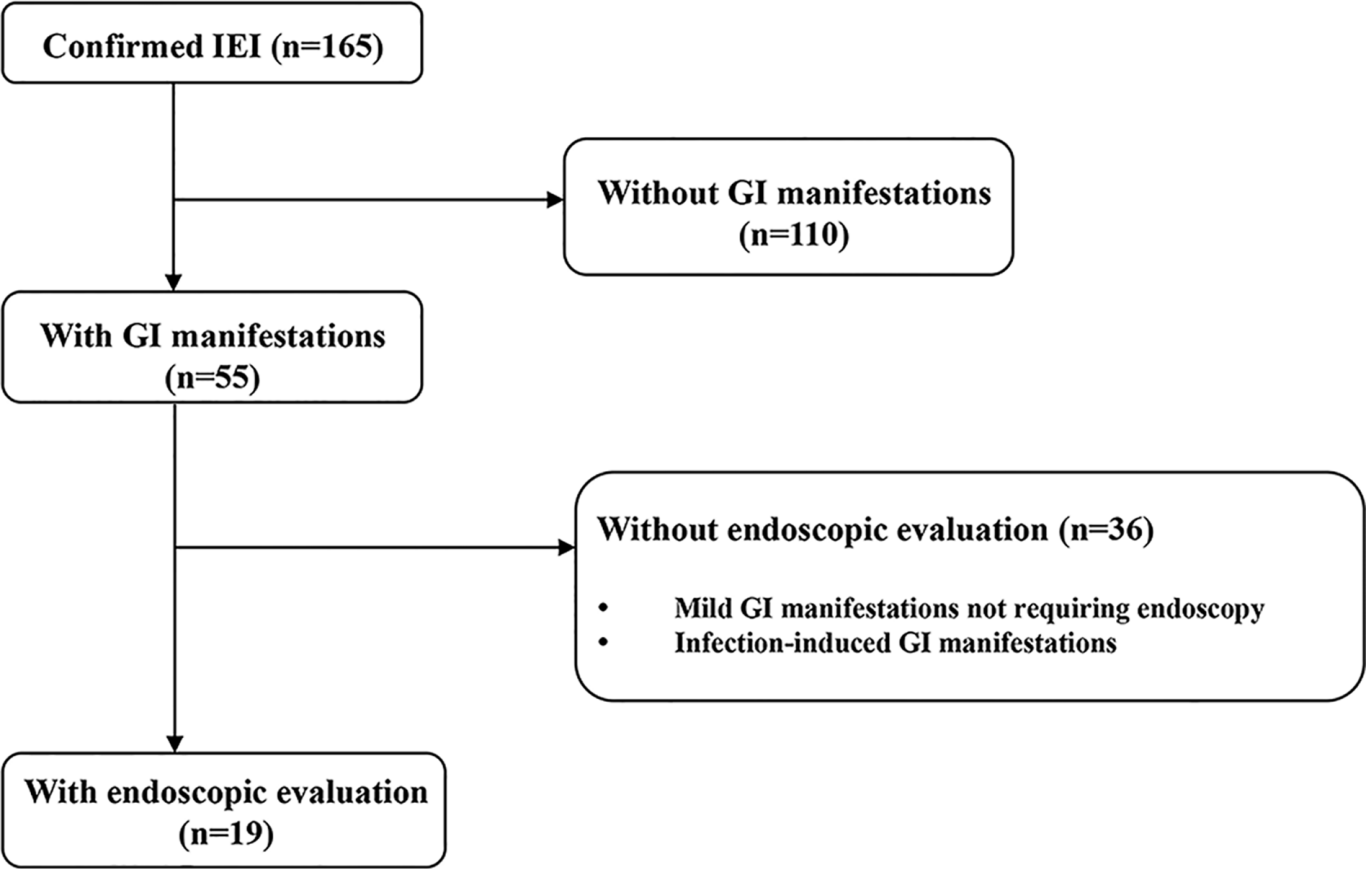
Study flow diagram. IEI, inborn errors of immunity; GI, gastrointestinal.

This was a retrospective cohort study conducted at the Department of Pediatrics of Samsung Medical Center from December 1995 to July 2020. Patients were eligible if they had IEI and developed GI manifestations that were severe enough to require endoscopic evaluation during the study period (**Figure 1**). Severe GI manifestations are GI bleeding, abdominal pain with signs or symptoms suggesting serious organic disease (e.g., weight loss, anorexia, anemia) associated with significant morbidity (e.g., prolonged hospitalization, limitation of usual activities), and chronic diarrhea of unexplained origin that lasts for more than 2–4 weeks. Cases with worsening GI symptoms that were caused by infection were excluded. The patients were divided according to the updated phenotypical classification for IEI (1), established by the International Union of Immunological Societies Expert Committee and were analyzed retrospectively. Baseline demographic and clinical data at initial endoscopy, including sex, age, growth indicators, GI manifestations, and other organ involvement in IEI were collected from electronic medical records. During the endoscopic evaluation, biopsies were taken from abnormal lesions to evaluate histopathologic alterations from the esophagus to the duodenum in esophagogastroduodenoscopy (EGD) and from the terminal ileum to rectum in ileocolonoscopy. For infants with severe GI manifestations, sigmoidoscopy was performed from the descending colon to rectum. Macroscopic and microscopic findings of the GI tract were analyzed. Continuous variables are presented as medians and interquartile ranges (IQRs) for non-normally distributed data or as means and standard deviations for normally distributed data. Categorical variables are presented as number and proportion. All statistical analyses were carried out using Rex (Version 3.0.3, RexSoft Inc., Seoul, Korea).

## Results

### Patient Characteristics

From December 1995 to July 2020, a total of 165 patients were diagnosed with IEI after immunological and/or genetic investigations. Amongst these 165 patients, the most common type of IEI is “Combined immunodeficiency with associated or syndromic features” (50/165, 30.3%), followed by “Predominantly antibody deficiencies” (34/165, 20.6%), “Congenital defects of phagocyte number or function” (30/165, 18.2%), “Diseases of immune dysregulation” (27/165, 16.4), “Defects in intrinsic and innate immunity” (7/165, 4.2%), “Complement deficiencies” (2/165, 1.2%), “Auto-inflammatory disorders” (1/165, 0.6%) and “Phenocopies of IEI” (1/165, 0.6%). Four patients were unidentified IEI (4/165, 2.4%).

Fifty-five patients (55/165, 33.3%) had GI manifestations, and 19 patients (19/165, 11.5%) received endoscopy. The most common type of IEI that involved severe GI symptoms was “Disease of immune dysregulation” (6/19, 31.6%) followed by “Phagocyte defects” (5/19, 26.3%) (**Table 1**). In 47.4% (9/19) of patients who were evaluated with endoscopy, severe and chronic GI symptoms preceded the diagnosis of IEI. Among the patients who developed GI manifestations before the diagnosis of IEI, the groups that accounted for the largest proportion was “Disease of immune dysregulation” (3/9, 33.3%) and “Phagocyte defects” (3/9, 33.3%). In particular, in patients who developed GI manifestations before IEI diagnosis, the average time from GI symptoms onset to IEI diagnosis was 2.6 years (range 0.2–14.4). Except for one patient, the diagnosis of IEI was not delayed as eight patients initially presented clinical symptoms similar to those of IBD.

**Table 1:** Distribution of IEI patients with severe GI symptoms enough to require endoscopy.

Group of Immunodeficiencies	Total, *n* (%)	Patients with GI symptoms	Patients with GI symptoms
		before IEI diagnosis, *n* (%)	after IEI diagnosis, *n* (%)
**Total**	**19 (100.0)**	**9 (47.4)**	**10 (52.6)**
**Predominantly antibody deficiencies**	**4 (21.1)**	**1 (5.3)**	**3 (15.8)**
Common variable immunodeficiency	2	0	2
Activated PI3k delta syndrome	2	1	1
**Congenital defects of phagocyte number or function**	**5 (26.3)**	**3 (15.8)**	**2 (10.5)**
Chronic granulomatous disease	4	2	2
Glycogen storage disease type 1b	1	1	0
**Disease of immune dysregulation**	**6 (31.6)**	**3 (15.8)**	**3 (15.8)**
CTLA4 deficiency	2	1	1
IPEX syndrome	1	1	0
XIAP	1	1	0
X-linked lymphoproliferative syndrome	2	0	2
**Defects in intrinsic and innate immunity**	**2 (10.5)**	**1 (5.2)**	**1 (5.3)**
Osteopetrosis	1	1	0
STAT1 gain of function	1	0	1
**Autoinflammatory disorders**	**2 (10.5)**	**1 (5.3)**	**1 (5.2)**
Blau syndrome	1	1	0
Chronic recurrent multifocal osteomyelitis	1	0	1

IEI, inborn errors of immunity; GI, gastrointestinal; PI3k, phosphoinositide 3 kinase; CTLA4, cytotoxic T lymphocyte-associated antigen-4; IPEX, immunodysregulation polyendocrinopathy enteropathy X-linked; XIAP, X-linked inhibitor of apoptosis; STAT1, signal transducer and activator of transcription 1.

Among patients who received endoscopy, thirteen patients (13/19, 68.4%) were male, and the mean age of all patients was 11.5 ± 7.9 years (range, 0.6–26.6) at the time of consultation and endoscopic evaluation. Patients presented with varied GI symptoms at the time of the initial endoscopic evaluation, including diarrhea (13/19, 68.4%), hematochezia (7/19, 36.8%), abdominal pain (6/19, 31.6%), perianal fistula and/or abscess (2/19, 10.5%) and recurrent oral ulcers (2/19, 10.5%). Furthermore, growth impairment was observed in six patients (6/19, 31.6%). In 10 patients who developed GI manifestations after the diagnosis of IEI, the elapsed time between the diagnosis of IEI and GI symptoms onset was 4.7 years (range 0.1–8.7 years). During the follow-up period, three patients (3/19, 15.8%) developed GI tract neoplasms (early gastric carcinoma, MALT (mucosa associated lymphoid tissue) lymphoma of colon and colonic tubular adenoma), and 12 patients (12/19, 63.2%) were diagnosed with IBD-like colitis.

Regarding therapies, patients diagnosed with activated PI3K δ syndrome (APDS), cytotoxic T lymphocyte-associated antigen 4 (CTLA 4) deficiency received therapies specifically oriented in the mechanism of the underlying immunological disease; sirolimus for APDS and CTLA4-Ig (abatacept) for CTLA 4 deficiency. Of two patients diagnosed with CTLA 4 deficiency, one received subtotal gastrectomy for early gastric carcinoma, and one received abatacept. In addition, six patients diagnosed with chronic granulomatous disease (CGD), glycogen storage disease (GSD) type 1b, immunodysregulation polyendocrinopathy enteropathy X-linked syndrome (IPEX syndrome), X-linked inhibitor of apoptosis protein deficiency (XIAP) underwent hematopoietic cell transplantation (HCT).

The patients with IBD-like colitis were treated with mesalazine (*n* = 8), azathioprine (*n* = 2), methotrexate (*n* = 2), steroids (*n* = 2), or anti-TNFα (*n* = 4) according to the IBD treatment guidelines (8). Three patients (CRMO, XIAP, and Osteopetrosis) responded to anti-TNFα treatment with IBD symptom improvement. Three patients (two patients with CGD, one patient with IPEX) underwent HCT to treat both IEI and IBD-like colitis. One patient with CTLA4 deficiency received abatacept and showed a dramatic response. Other baseline characteristics of the study subjects are summarized in **Table 2** and detailed clinical characteristics of 19 patients are presented in **Table 3**.

**Table 2:** Baseline characteristics of IEI patients with GI manifestations required endoscopic evaluation.

	*Total (n = 19)*
Males, *n* (%)	13 (68.4)
Age at diagnosis *of* IEI, *years* (IQR)	5.1 (1.4, 10.8) (range, 0.4–24.5)
Age at initial GI symptoms, *years*	9.3 ± 7.0 (range, 0–24.9)
Age at initial endoscopy, *years*	11.5 ± 7.9 (range, 0.6–26.6)
GI manifestations before IEI diagnosis, *n* (%)	9 (47.4)
Observational duration, years (IQR)	5.1 (1.5–11.4)
Body weight z-score	-1.3 ± 1.5
Height z-score	-1.1 ± 1.3
BMI z-score	-0.6 ± 1.8
Growth impairment, *n* (%)	6 (31.6)
GI manifestations, n (%)	
Abdominal pain	6 (31.6)
Diarrhea	13 (68.4)
Hematochezia	7 (36.8)
Recurrent oral ulcer	2 (10.5)
Perianal fistula/abscess	2 (10.5)
Premalignant and malignant lesions during follow-up	3 (15.8)
IBD-like colitis, *n* (%)	12 (63.2)
Other organ involvement*, *n* (%)	
Lung	6 (31.6)
Skin	4 (21.1)
Eye	2 (10.5)
Kidney	2 (10.5)
Liver	2 (10.5)
Heart	1 (5.3)

IEI, inborn errors of immunity; GI, gastrointestinal; IQR, interquartile range; BMI, body mass index; IBD, inflammatory bowel disease.

**Table 3:** Clinical characteristics of 19 patients diagnosed with inborn errors of immunity who required endoscopic evaluations.

No.	Diagnosis	Gene	Sex	Age at GI symptoms onset (yrs)	GI manifestations	Other involved organ	IBD-like colitis	Neoplasms (premalignant & malignant lesions)	Treatment for GI symptoms
1	CVID	N/A	Female	0.5	Diarrhea, Growth retardation	Liver, Kidney, Lung, Heart	–	Colonic tubular adenoma	Steroid
2	CVID	N/A	Female	16.3	Diarrhea, Abdominal pain,	Lung	+ (UC)	–	Mesalazine
					Growth retardation				
3	APDS	PI3K mutation	Female	5.6	Hematochezia	Lung, Lymph node	–	MALT lymphoma	Sirolimus
4	APDS	PI3K mutation	Male	4.2	Hematochezia	Salivary gland	–	–	Sirolimus
5	CGD	CYBB mutation	Male	0.1	Diarrhea, Growth retardation,	None	+ (CD)	–	Scheduled for HCT
					Perianal abscess				
6	CGD	CYBB mutation	Male	9.3	Diarrhea, Hematochezia, Growth retardation, Oral ulcer	Lung, Eye	+ (UC)	–	Mesalazine
7	CGD	CYBB mutation	Male	8.5	Oral ulcer	None	+ (CD)	–	Mesalazine
8	CGD	CYBB mutation	Male	2.5	Diarrhea, Hematochezia	None	+ (CD)	–	Steroid, HCT
9	GSD type Ib	SLC37A4 mutation	Male	2.6	Diarrhea, Growth retardation	None	+ (CD)	–	Mesalazine, HCT
10	CTLA4 deficiency	CTLA4 mutation	Female	18.4	Diarrhea	Lung, Skin	–	Early gastric carcinoma	Subtotal gastrectomy
11	CTLA4 deficiency	CTLA4 mutation	Female	10.0	Diarrhea, Hematochezia,	Lung, Eye, Kidney, Liver, Skin, Salivary gland	+ (UC)	–	Mesalazine, Abatacept
12	IPEX	FOXP3 mutation	Male	8.3	Diarrhea, Hematochezia, Abdominal pain	None	+ (CD)	–	MTX, Adalimumab, HCT
13	XIAP	XIAP mutation	Male	8.4	Diarrhea, Abdominal pain,	Skin	+ (CD)	–	Mesalazine,Adalimumab, Infliximab
					Growth retardation				
14	XLP	SH2D1A mutation	Male	10.7	Incidental finding of malignancy	None	–	–	HCT
15	XLP	SH2D1A mutation	Male	1.2	Hematochezia	None	–	–	HCT
16	Osteopetrosis	CLCN7 mutation	Male	17.5	Diarrhea, Abdominal pain,	None	+ (UC)	–	Azathioprine, Mesalazine, Infliximab
					Perianal fistula				
17	STAT1 GOF	STAT1 mutation	Female	24.8	Abdominal pain	Lung, Skin	–	–	Prokinetics
18	Blau syndrome	NOD2 mutation	Male	0.5	Diarrhea	Skin	+ (CD)	–	Methotrexate, Steroid
19	CRMO	N/A	Male	13.9	Diarrhea, Abdominal pain,	Skin, Bone (costovertebral junction, femur, patella, tibia, talus)	+ (CD)	–	Azathioprine, Mesalazine, Infliximab
					Growth retardation				

GI, gastrointestinal; IBD, inflammatory bowel disease; CVID, common variable immunodeficiency; N/A, non-acting; UC, ulcerative colitis; APDS, activated PI3k delta syndrome; PI3K, phosphorinositide 3 kinase; MALT lymphoma, mucosa associated lymphoid tissue lymphoma; CGD, chronic granulomatous disease; CYBB, cytochrome b-245 β chain; CD, Crohn’s disease; HCT, hematopoietic cell transplantation; GSD, glycogen storage disease; SLC, solute carrier; CTLA4, cytotoxic T lymphocyte-associated antigen-4; IPEX, immunodysregulation polyendocrinopathy enteropathy X-linked; FOXP3, Forkhead box protein P3; MTX, methotrexate; XIAP, X-linked inhibitor of apoptosis; XLP, X-linked lymphoproliferative syndrome; SH2D1A, SH2 domain containing 1A; CLCN7, chloride voltage-gated channel 7; STAT1, signal transducer and activator of transcription 1; GOF, gain of function; NOD2, nucleotide binding oligomerization domain containing 2; CRMO, Chronic recurrent multifocal osteomyelitis.

### Endoscopic Findings

**Figure 2:**
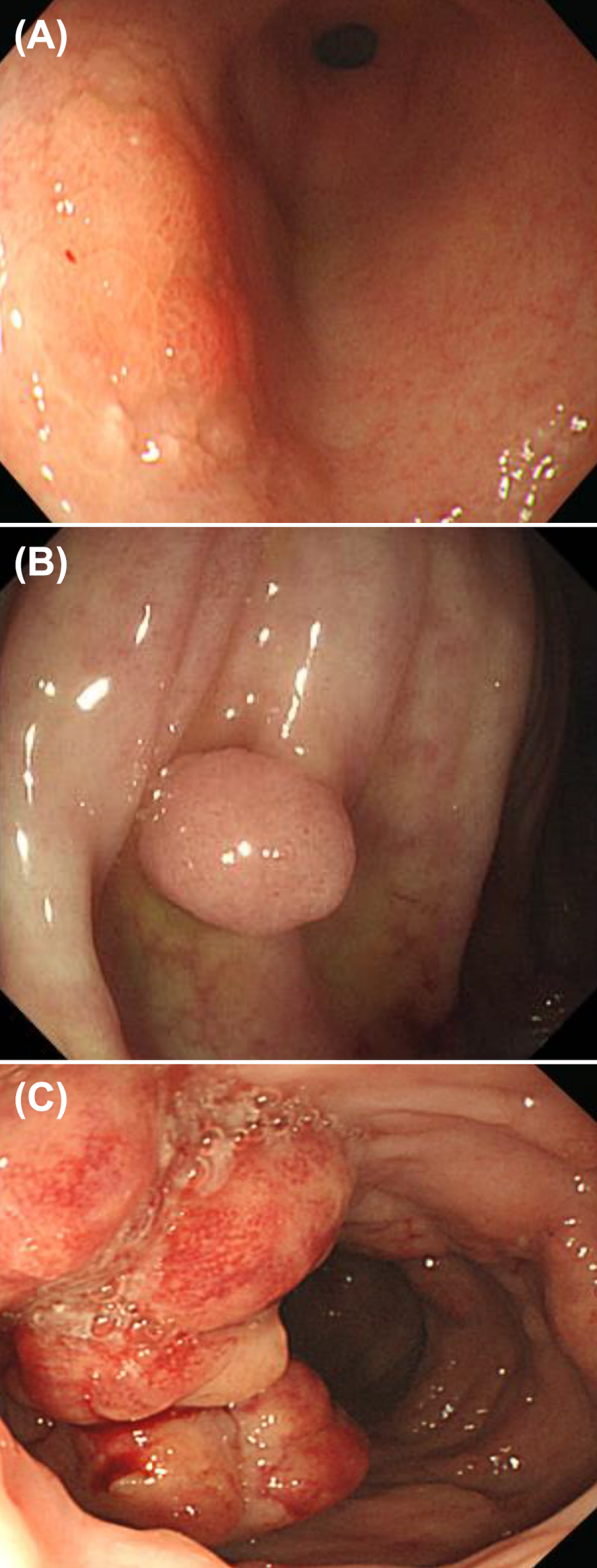
Gastrointestinal tract malignancy in patients with primary immunodeficiency **(A)** Early gastric carcinoma in a patient with CTLA4 deficiency. **(B)** Colonic tubular adenoma in a patient with common variable antibody deficiency. **(C)** Colonic MALT lymphoma in a patient with activated PI3Kδ syndrome.

Nineteen patients with IEI underwent a total of 111 endoscopies during the follow-up period. These included 57 EGDs, 53 ileocolonoscopies and one sigmoidoscopy. **Table 4** gives the details of the endoscopic findings of all 19 patients. Endoscopic examination of the esophagus was normal in 14 patients (14/19, 73.7%), and five patients exhibited abnormality in the esophagus such as esophagitis, esophageal ulcer, and esophageal varix. Chronic gastritis (8/19, 42.1%) was the most common macroscopic finding in the stomach, and early gastric carcinoma was diagnosed in one patient with CTLA 4 deficiency (**Figure 2A**). Duodenal ulcer and lymphoid hyperplasia of the duodenum were each identified in four patients (4/19, 21.1%).

**Table 4:** Endoscopic findings in patients with inborn errors of immunity.

	Endoscopic Findings (*n* = 19)	Predominantly antibody deficiencies (*n* = 4)		Congenital defects of phagocyte number or function (*n* =5)		Defects in intrinsic and innate immunity (*n* = 2)		Disease of immune dysregulation (*n* = 6)				Auto-inflammatory Disorders (*n* = 2)		
		CVID	APDS	CGD	GSD type 1b	Osteopetrosis	STAT1 GOF	IPEX	XIAP	CTLA4 deficiency	XLP	Blau syndrome	CRMO	
**Esophagus**	Esophagitis	–	–	1/4^e^	–	–	1/1	–	–	1/2^i^	–	–	–	
	Esophageal ulcer	–	–	1/4^e^	–	–	1/1	–	–	1/2^i^	–	–	–	
	Esophageal varix	1/2^a^	–	–	–	–	–	–	–	1/2^j^	–	–	–	
**Stomach**	Chronic gastritis	1/2^b^	1/2^c^	1/4^f^	-	1/1	-	-	1/1	2/2^i,j^	1/2^k^	-	-	
	Gastric ulcer	1/2^a^	-	-	-	-	-	-	-	1/2^i^	-	1/1	-	
	Early gastric cancer	-	-	-	-	-	-	-	-	1/2^i^	-	-	-	
**Duodenum**	Duodenitis	–	–	–	–	–	–	–	1/1	–	–	–	–	
	Duodenal ulcer	1/2^a^	–	–	–	–	–	–	1/1	1/2^i^	–	1/1	–	
	Lymphoid hyperplasia	1/2^b^	2/2^c,d^	–	–	–	–	–	–	1/2^i^	–	–	–	
**Ileum**	Ileitis	-	1/2^d^	1/4^e^	1/1	1/1	-	-	-	-	-	1/1	-	
	Atrophic change	-	-	-	-	-	-	-	-	1/2^j^	-	-	-	
	Lymphoid hyperplasia	2/2^a,b^	2/2^c,d^	-	-	-	-	-	1/1	1/2^j^	-	-	-	
**Colon**	Lymphoid hyperplasia	1/2^b^	1/2^c^	1/4^h^	–	–	–	–	–	–	–	–	–	
	Mucosal edema	1/2^b^	–	1/4^f^	1/1	–	–	1/1	1/1	1/2^j^	–	–	1/1	
	Ulcer	1/2^b^	–	3/4^e,f,g^	1/1	1/1	–	1/1	1/1	1/2^j^	1/2 ^k^	–	1/1	
	Adenoma	1/2^a^	–	–	–	–	–	–	–	–	–	–	–	

*Each alphabet represents one patient.

CVID, common variable immunodeficiency; APDS, activated PI3k delta syndrome; CGD, chronic granulomatous disease; GSD, glycogen storage disease; STAT1, signal transducer and activator of transcription 1; GOF, gain of function; IPEX, immunodysregulation polyendocrinopathy enteropathy X-linked; XIAP, X-linked inhibitor of apoptosis; CTLA4, cytotoxic T lymphocyte-associated antigen 4; XLP, X-linked lymphoproliferative syndrome; CRMO, chronic recurrent multifocal osteomyelitis.

On ileocolonoscopy, premalignant and malignant lesions were found in the lower GI tracts of two patients: colonic tubular adenoma in a common variable immunodeficiency (CVID) patient and MALT lymphoma in an APDS patient proved by immunoglobulin heavy chain gene rearrangement (**Figures 2B, C**).

**Figure 3:**
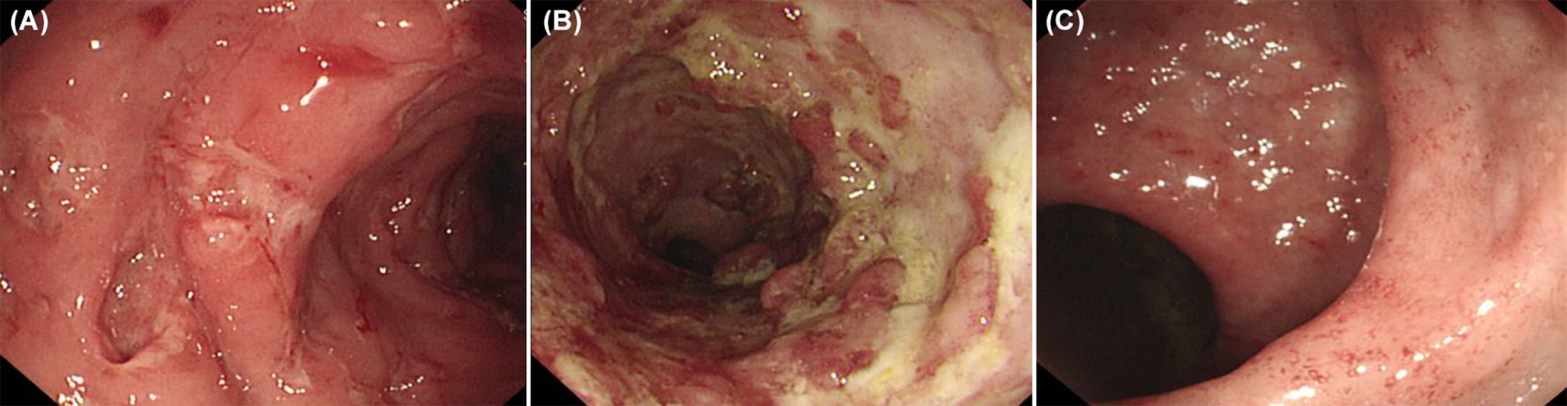
Inflammatory bowel disease like colitis in patients with primary immunodeficiency. **(A)** Multiple large deep ulcers on whole colon in a patient with X-linked inhibitor of apoptosis protein deficiency. **(B)** Longitudinal large deep ulcer on whole colon in a patient with Chronic recurrent multifocal osteomyelitis. **(C)** Mucosal edema, tiny superficial ulcer, loss of vascularity on colon in patients with osteopetrosis.

Features of IBD-like colitis were found in 12 patients (12/19, 63.2%): five patients were diagnosed with congenital defects of phagocyte number or function (CGD, GSD type 1b); three patients with diseases of immune dysregulation (IPEX syndrome, XIAP deficiency (**Figure 3A**), CTLA4 deficiency); two patients with auto-inflammatory disorders [Blau syndrome; chronic recurrent multifocal osteomyelitis, CRMO (**Figure 3B**)]; one patient with defects in intrinsic and innate immunity [osteopetrosis (**Figure 3C**)]; and one patient with predominantly antibody deficiencies (CVID).

**Figure 4:**
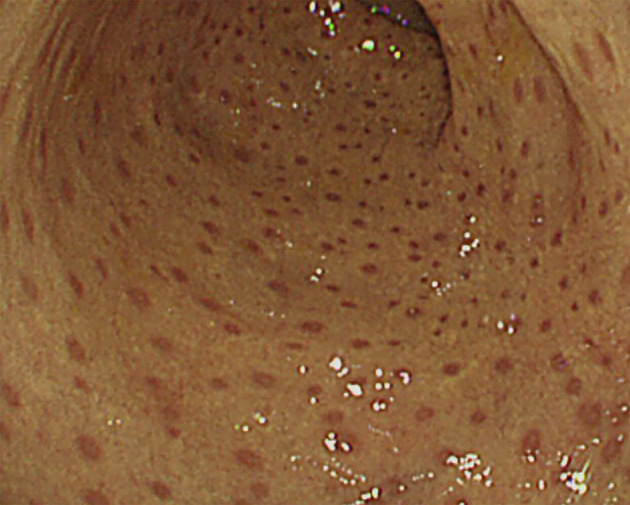
Colonoscopic ‘leopard sign’ in Chronic granulomatous disease. Endoscopic view of the colonic mucosae showing the leopard sign appearing as brown dots distributed across a yellowish edematous mucosa.

One out of four patients with CGD showed colonic leopard sign appearing as brown dots distributed across a yellowish edematous mucosa which means microscopically that aggregation of pigment-laden macrophages on the mucosa (**Figure 4**).

### Histopathologic Findings

**Figure 5:**
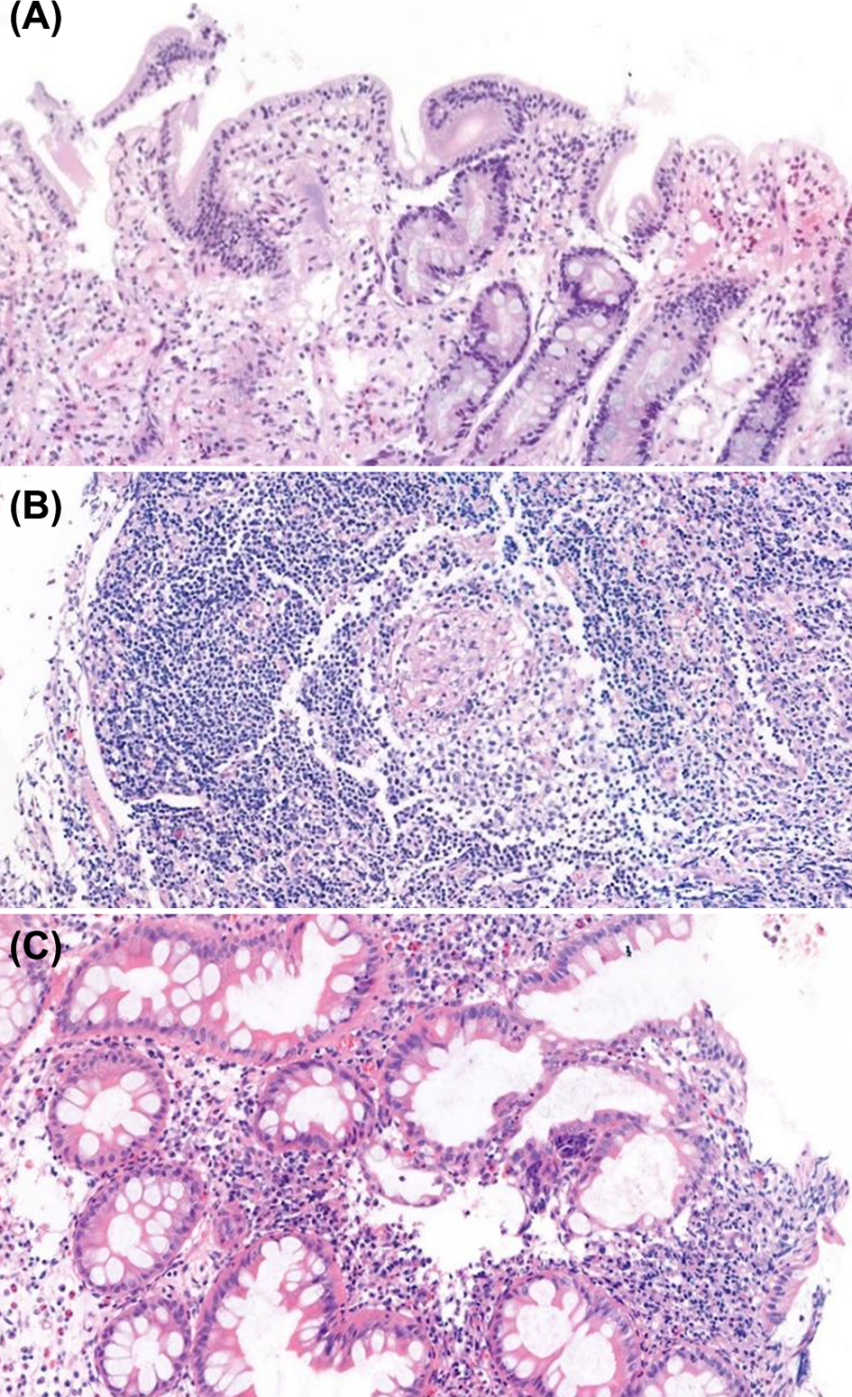
Histopathologic findings of endoscopic biopsies (H&E staining). **(A)** Duodenal biopsy with atrophy of villi, apoptotic bodies and increased intraepithelial lymphocytes, consistent with common variable immunodeficiency. **(B)** Colonic biopsy with non-caseating granuloma in patient with X-linked inhibitor of apoptosis protein syndrome. **(C)** Colonic biopsy with cryptitis and crypt distortion in patient with osteopetrosis.

The histopathologic findings of the study subjects are depicted in **Table 5**. Mucosal ulcer and inflammation were commonly observed endoscopic pathologies in all patients. All CVID patients were found to have intraepithelial lymphocytosis (IEL) of the GI tact, and one patient showed apoptosis and atrophy of duodenal villi (**Figure 5A**). Of note, this patient had severe profuse diarrhea due to a norovirus infection that led to significant weight loss. Likewise, all APDS patients showed IEL in the GI tract. Twelve patients (12/19, 63.2%) were found to have IBD features on biopsies taken from the GI tract. Eight patients had histopathologic features of Crohn’s disease such as active inflammation and non-caseating granuloma (**Figure 5B**); they were diagnosed with CGD, GSD type 1b, IPEX, XIAP, Blau syndrome and CRMO. Four patients (CVID, APDS, CGD, and Osteopetrosis) had features of ulcerative colitis such as crypt distortion and cryptitis/crypt abscess (**Figure 5C**).

**Table 5:** Histopathologic findings of GI tract in patients with inborn errors of immunity.

	Histopathologic Findings (*n* = 19)		Predominantly antibody deficiencies (*n* = 4)		Congenital defects of phagocyte number or function (*n* =5)		Defects in intrinsic and innate immunity (*n* = 2)		Disease of immune dysregulation (*n* = 6)				Autoinflammatory Disorders (*n* = 2)	
			CVID	APDS	CGD	GSD type 1b	Osteopetrosis	STAT1 GOF	IPEX	XIAP	CTLA4 deficiency	XLP	Blau syndrome	CRMO
**Esophagus**		Esophagitis	–	–	1/4^e^	N/A	–	–	–	–	1/2^h^	–	–	–
		Esophageal ulcer	–	–	1/4^e^		–	1/1	–	–	–	–	–	–
		Eosinophilic infiltration	1/2^a^	–	–		–	–	–	–	–	–	–	–
**Stomach**		H.pylori (-) gastritis	1/2^b^	1/2^c^	1/3^f^	N/A	1/1	-	-	1/1	2/2^h,i^	1/2 ^j^	1/1	-
		H.pylori (+) gastritis	-	1/2^d^	-		-	-	-	-	-	-	-	-
		Intestinal metaplasia	-	-	-		-	-	-	-	1/2^h^	-	-	-
		Tubular adenocarcinoma	-	-	-		-	-	-	-	1/2 ^h^	-	-	-
**Duodenum**		Duodenitis	–	1/2^d^	–	N/A	–	–	–	1/1	–	–	1/1	–
		Apoptosis	1/2^a^	–	–		–	–	–	–	–	–	–	–
		Atrophy of villi	1/2^a^	–	–		–	–	–	–	–	–	–	–
		Intraepithelial lymphocytosis	1/2^a^	–	–		–	–	–	–	–	–	–	–
**Ileum**		Ileitis	-	-	1/1	1/1	-	-	-	-	-	-	1/1	1/1
		Intraepithelial lymphocytosis	1/2^a^	2/2^c,d^	-	-	-	-	-	-	-	-	-	-
		Lymphoid hyperplasia	-	2/2^c,d^	-	-	-	-	-	-	1/2^i^	-	-	-
		Eosinophilic infiltration	-	1/2^d^	-	-	-	-	-	-	1/2^h^	-	-	-
**Colon**		Active inflammation	1/2^b^	1/2^c^	3/3^e,f,g^	1/1	–	–	1/1	1/1	1/2^i^	1/2 ^j^	1/1	1/1
		Apoptosis	1/2^b^	–	–	–	–	–	–	–	–	–	–	–
		Pigmented macrophage	–	–	1/3^g^	–	–	–	–	–	–	–	–	–
		Crypt distortion	–	–	1/3^f^	–	1/1	–	–	–	–	–	–	–
		Cryptitis/Crypt abscess	1/2^b^	–	1/3^f^	–	1/1	–	–	–	1/2^i^	–	–	1/1
		Non-caseating granuloma	–	–	1/3^f^	1/1	–	–	1/1	1/1	–	–	–	–
		Intraepithelial lymphocytosis	1/2^b^	2/2^c,d^	1/3^f^	–	–	–	–	–	1/2^i^	–	–	–
		Eosinophilic infiltration	–	1/2^d^	–	–	–	–	–	–	1/2^i^	–	–	–
		Tubular adenoma	1/2^a^	–	–	–	–	–	–	–	–		–	–
		MALT lymphoma	–	1/2^c^			–	–	–	–	–		–	–

*Each alphabet represents one patient.

CVID, common variable immunodeficiency; APDS, activated PI3k δ syndrome; CGD, chronic granulomatous disease; GSD, glycogen storage disease; STAT1, signal transducer and activator of transcription 1; GOF, gain of function; IPEX, immunodysregulation polyendocrinopathy enteropathy X-linked; XIAP, X-linked inhibitor of apoptosis; CTLA4, cytotoxic T lymphocyte-associated antigen-4; XLP, X-linked lymphoproliferative syndrome; CRMO, chronic recurrent multifocal osteomyelitis; MALT lymphoma, mucosa associated lymphoid tissue lymphoma.

## Discussion

Few studies analyzing the GI tract diseases in IEI have been conducted to date. In addition, there is little data on endoscopic and histopathologic findings of the GI tract in IEI patients (9–13). With increasing awareness, IEI is an emerging disease in Asia, however, there are scarce data on IEI including South Korea (14). This is the first study to observe the clinical, endoscopic, and histopathologic findings of the GI tract in IEI patients in a single center over the long term.

Depending on the nature of the specific type of IEI, the frequency of GI manifestations can range from 5 to 50% (6). In our cohort, 33.3% of patients (55/165) had GI manifestations, and 11.5% of all IEI patients (19/165) had severe GI manifestations that required endoscopy. These frequencies are consistent with previously reported studies (6). In our subjects, diarrhea was the most common symptom (13/19, 68.4%), followed by hematochezia, abdominal pain and growth impairment, which were previously reported as prevalent GI symptoms in IEI patients (7, 9, 15–22). It is already known that growth impairment was frequently detected, and the risk of growth failure is four times higher than in the healthy pediatric population (23–25). Growth can be affected in a variety of ways in patients with IEI: hyper catabolic states such as recurrent inflammatory and/or infectious conditions; reduced caloric intake because of low ingestion or malabsorption; endocrine disorders; osteoarticular dysplasia; and genetic syndromes such as Kabuki syndrome (26). In the studies regarding to patients with IEI, growth impairment was found in 11–28% of patients (27, 28). In this study, the proportion of patients with growth impairment was higher than in previous studies (6/19, 31.6%), which may be due to a cohort of patients with severe GI symptoms requiring endoscopies.

Malignancy is the second most prevalent cause of mortality in patients with IEI, after infection (29). The lifetime risk of cancer in children with IEI is estimated to be 5–25% (30, 31), and the relative risk of cancer in patients with IEI is 1.4 to 1.6 times greater than that in the general population (32, 33). It has been reported that genomic instability due to defective DNA repair processes, lack of control of chronic inflammation, susceptibility to oncogenic viruses (such as human papillomavirus or Epstein-Barr virus) and other unknown mechanisms increase the risk of malignancies (29, 34, 35).

The GI tract malignancy may be diagnosed at initial diagnosis of IEI or during a follow-up visit in a patient with known IEI. Within the USIDNET cohort, 171 different cancers were reported, of which GI cancer accounted for 8%. In our cohort, 1.8% of patients with IEI (3/165) were diagnosed with GI tract neoplasms (premalignant and malignant lesions) during the study period: early gastric carcinoma in CTLA 4 deficiency, MALT lymphoma in APDS, and colonic tubular adenoma in CVID. However, in patients who underwent endoscopic evaluation for severe GI symptoms, the rate of GI tract neoplasms increased up to 15.8% (3/19).

Currently, there are no specific recommendations for cancer screening in high-risk IEI individuals, and the prerequisites listed in screening protocols differ from country to country because of variations in cancer incidence, mortality rates, racial differences, and socio-economic states. In case of one of patient with APDS who developed MALT lymphoma, the patient had severe hematochezia required initial colonoscopy which showed only adenomatous changes in the colon at 6 years old. Therefore, colonoscopy was performed regularly thereafter. Five years later (11 years old), multi-lobulated masses were observed in the colon which revealed MALT lymphoma for which careful observation was continued. Later, the patient’s genetic study revealed the diagnosis of APDS and sirolimus was started. MALT lymphomatous lesions disappeared six months after the initiation of sirolimus. In a CTLA 4 deficiency patient, gastric adenoma was diagnosed at the age of 19 and progressed to gastric adenocarcinoma six years later which required surgical resection. Clinicians should make sure not to miss routine assessments that can identify the cause of GI symptoms such as cancer, and radiologists and pathologists should be aware of the manifestations of IEI when interpreting test results. In addition, it is important to ensure that patients and their parents are aware of the possibility of malignancies and that they know to report any changes in their health status to their physicians.

With the recent advances in diagnostic and therapeutic strategies for IEI, the lifespan of patients is increasing at a remarkable rate, and inflammatory and autoimmune manifestations are emerging as an important issue. Immune dysregulation in IEI leads to autoimmune manifestations by affecting variable immune pathways such as T cell development/tolerance, T cell signaling, interferon signaling pathway and/or resolution of inflammatory (36). In addition, the influence of chronic and recurrent infection through suboptimal and chronic immune response, bystander activation, super antigens activation and molecular mimicry is thought to play an important role in the development of autoimmunity (37). For example, Broides et al. reported that non-infectious colitis is common, and elevation of fecal calprotectin, which is a quantitative measure of intestinal inflammation and indicates the presence of inflammation in the GI tract, is common in asymptomatic CGD patients without overt GI manifestations (38).

In the same vein, IEI is a rare but important cause of IBD, especially in very early onset IBD (39). IBD is a multifactorial idiopathic chronic inflammatory disease of the GI tract caused by a dysregulated immune response to host intestinal microbiota (40, 41), and may be the first or only symptom of underlying IEI. IBD-like colitis in IEI can be further understood through the lens of IEI-associated autoimmunity to an idiopathic autoimmune and inflammatory condition. As described above, 63.2% (12/19) of patients in our cohort also showed inflammatory and autoimmune features in the GI tract. These patients showed endoscopic and histopathologic features of IBD and were treated according to the IBD guideline (8). If IEI-associated IBD is suspected, early immunologic tests should be performed. The types of IEI related to IBD are consistent with genetic defects underlying IEI that are known to be associated with autoimmunity: CGD, X-linked proliferative disease, IPEX, CTLA4 deficiency, and CVID (36).

The diagnosis of underlying IEI has an important impact on the therapeutic decisions that are made in cases of IBD. Some treatments for certain types of IEI have been found to be effective against IBD. For example, abatacept is effective in patients with CTLA4 deficiency, as seen in a patient in our study, and HCT is known as a therapeutic option for treatment of XIAP, CGD, and IL-10 deficiency and associated IBD-like colitis. Early diagnosis and treatment of IEI-associated IBD is critical to improving disease outcomes and preventing and mitigating severe complications. Endoscopy may be helpful in diagnosing IEI-induced GI manifestations, assessing the severity of the disease, and monitoring the response to medical treatment (9). The GI tracts of patients with IEI shows a broad spectrum of histopathologic patterns. The condition can mimic lymphocytic gastroenteritis, granulomatous disease, acute graft-versus-host disease (GVHD), and IBD (2). The histopathologic findings in our study showed that features of GVHD as apoptosis and prominent lymphocytosis, features of celiac disease as villous blunting with IEL, and features of IBD as cryptitis, crypt distortion or non-caseating granuloma. From this point of view, pathologists should remember that certain features of IBD may appear in IEI patients. Although the characteristic feature of IEI is increased susceptibility to infection, many cases are associated with GI disease and initially present with GI symptoms, meaning that a routine evaluation of the GI tract is necessary. A history of recurrent infections, atypical clinical and/or histologic features of an uncommon pattern of GI disease, or a poor response to conventional therapy should facilitate further immunologic evaluation.

In conclusion, GI manifestations may appear concurrently with the onset of IEI or occur during the course of IEI. In addition, it is necessary to always consider that autoimmune/inflammatory diseases and malignancies may occur in patients who have already been diagnosed with IEI. An investigation of immunodeficiency in patients with atypical GI symptoms can lead to a correct diagnosis and appropriate disease-specific therapy. Thus, there is a need for increased awareness of GI manifestations in IEI. Gastroenterologists and immunologists should consider endoscopy when atypical and/or refractory GI manifestations appear in IEI patients. A collaborative approach among gastroenterologists and immunologists in evaluating IEI patients with refractory GI symptoms is required to better understand the full spectrum of GI tract diseases and associated complications. Also, when physicians in various fields, such as radiologists and pathologists, increase their awareness of IEI-specific complications, early diagnosis, and disease-specific treatment for IEI can be made more appropriately.

## Data Availability Statement

The raw data supporting the conclusions of this article will be made available by the authors, without undue reservation.

## Ethics Statement

The studies involving human participants were reviewed and approved by Institutional Review Board of Samsung Medical Center (IRB File No. 2021-01-067). Written informed consent to participate in this study was provided by the participants’ legal guardian/next of kin.

## Author Contributions

Guarantor of the article: ESK. ESK contributed in the conception and design of the study, acquisition, analysis and interpretation of data, drafting of the initial manuscript, and critical revision for important intellectual content. DK, YY, YK and SP contributed to the acquisition, analysis and interpretation of data. JK, KMA and SA contributed to the acquisition and interpretation of data. YHC contributed to the conception and design of the study and critical revision for important intellectual content. Y-JK contributed to the conception and design of the study, analysis and interpretation of data, drafting of the initial manuscript and critical revision for important intellectual content. MJK contributed to the conception and design of the study, interpretation of data, drafting of the initial manuscript, and critical revision for important intellectual content. All authors contributed to the article and approved the submitted version

## Conflict of Interest

The authors declare that the research was conducted in the absence of any commercial or financial relationships that could be constructed as a potential conflict of interest.

## Publisher’s Note

All claims expressed in this article are solely those of the authors and do not necessarily represent those of their affiliated organizations, or those of the publisher, the editors and the reviewers. Any product that may be evaluated in this article, or claim that may be made by its manufacturer, is not guaranteed or endorsed by the publisher.
